# Inflammatory stress in SARS-COV-2 associated Acute Kidney Injury

**DOI:** 10.7150/ijbs.58791

**Published:** 2021-04-10

**Authors:** Junzhe Chen, Wenbiao Wang, Ying Tang, Xiao-ru Huang, Xueqing Yu, Hui-Yao Lan

**Affiliations:** 1 Departments of Medicine & Therapeutics, Li Ka Shing Institute of Health Sciences, and Lui Che Woo Institute of Innovative Medicine, The Chinese University of Hong Kong, Hong Kong, China.; 2 Department of Nephrology, The Third Affiliated hospital, Southern Medical university, Guangzhou, China.; 3 Guangdong Key Laboratory of Virology, Institute of Medical Microbiology, Jinan University, Guangzhou, China.; 4 Guangdong-Hong Kong Joint Laboratory for Immunity and Genetics of Chronic Kidney Disease, Guangdong Academy of Medical Science, Guangdong Provincial People's Hospital, Guangzhou, China.; 5 Guangdong-Hong Kong Joint Laboratory for Immunity and Genetics of Chronic Kidney Disease, The Chinese University of Hong Kong, Hong Kong, China.

**Keywords:** COVID-19, AKI, cytokines, inflammation, mechanisms

## Abstract

Increasing clinical evidence shows that acute kidney injury (AKI) is a common and severe complication in critically ill COVID-19 patients. The older age, the severity of COVID-19 infection, the ethnicity, and the history of smoking, diabetes, hypertension, and cardiovascular disease are the risk factor for AKI in COVID-19 patients. Of them, inflammation may be a key player in the pathogenesis of AKI in patients with COVID-19. It is highly possible that SARS-COV-2 infection may trigger the activation of multiple inflammatory pathways including angiotensin II, cytokine storm such as interleukin-6 (IL-6), C-reactive protein (CRP), TGF-β signaling, complement activation, and lung-kidney crosstalk to cause AKI. Thus, treatments by targeting these inflammatory molecules and pathways with a monoclonal antibody against IL-6 (Tocilizumab), C3 inhibitor AMY-101, anti-C5 antibody, anti-TGF-β OT-101, and the use of CRRT in critically ill patients may represent as novel and specific therapies for AKI in COVID-19 patients.

## Introduction

COVID-19 is a progressive viral pneumonia with a broad spectrum of clinical manifestations, ranging from asymptomatic to mild (80%), severe (10-15%) or critical and death (2-5%) 1, 2. Among critically ill COVID-19 patients, acute respiratory distress syndrome (ARDS) and multiorgan failure including acute kidney injury (AKI) are the most common co-morbidities 3-5. In this review article, we are focusing on SARS-CoV-2-associated AKI. The possible mechanisms and pathways related to SARS-CoV-2-associated AKI are discussed.

## Epidemiology of AKI in COVID-19 patients

Increasing evidence shows that there is high prevalence of AKI in COVID-19 patients 6, 7. The manifestations of AKI are diverse, from proteinuria, hematuria, elevated serum creatinine (Scr) or blood urea nitrogen (BUN) levels to acute renal failure. A meta-analysis shows that more than half (57%) of COVID-19 patients develop proteinuria, accompanied by elevated serum levels of Scr (9.6%-15.5%) and BUN (13.7-14.1%) 5, 6. The CT scan also shows renal inflammation and edema 8. Pathologically, diffuse proximal tubule injury with loss of brush border and frank necrosis is found in COVID-19 patients with AKI 9, 10.

Compared to patients with Severe Acute Respiratory Syndrome (SARS) and Middle East Respiratory Syndrome (MERS) in which the incidence of AKI is 6.7% and 42% respectively 11, 12. The incidence of AKI in COVID-19 patients is highly variable. In the early reports from China, COVID-19 patients with AKI was rare 13, 14, but increased to 10% in a later study 15, and became more severe with the incident rate of 25%-29% in those admitted to ICU 16, 17. The large cohort studies in the western countries revealed that the incidence of AKI was 27%-37% 18, 19 and became more severe (68%) in critically ill COVID-19 patients who were admitted to ICU in the New York city 20. Nevertheless, it is now clear that the incidence of AKI in COVID-19 patients is associated with the age, smoking, the cytokine storm, the severity of disease, the ethnicity, and the history of diabetes, hypertension, and cardiovascular disease 7. Thus, AKI is an independent risk factor for the poor long-term renal outcome and mortality in critically ill COVID-19 patients 21, 22. During the follow-up study, AKI is a major cause of in-hospital mortality. In addition, the complete kidney recovery rate of AKI in COVID-19 infection is only about 30-45% based on the recent reports 15, 20, 23. Thus, AKI is one of severe complications and mortality of in-hospital COVID-19 patients, however, mechanisms of COVID-19-associated AKI remain largely unclear and need further studies.

## Inflammation may be a mechanism of AKI in COVID-19 patients

Multiple factors such as direct virus infection, cytokine storm, hypoxia, sepsis shock, hemodynamic instability and rhabdomyolysis, hypertension, and diabetes may be associated with AKI in COVID-19 patients. Of these factors, inflammation stress may be a mechanism of AKI in COVID-19 patients, which is discussed below.

### Angiotensin II (Ang II) and hypertensive stress

**Figure 1:**
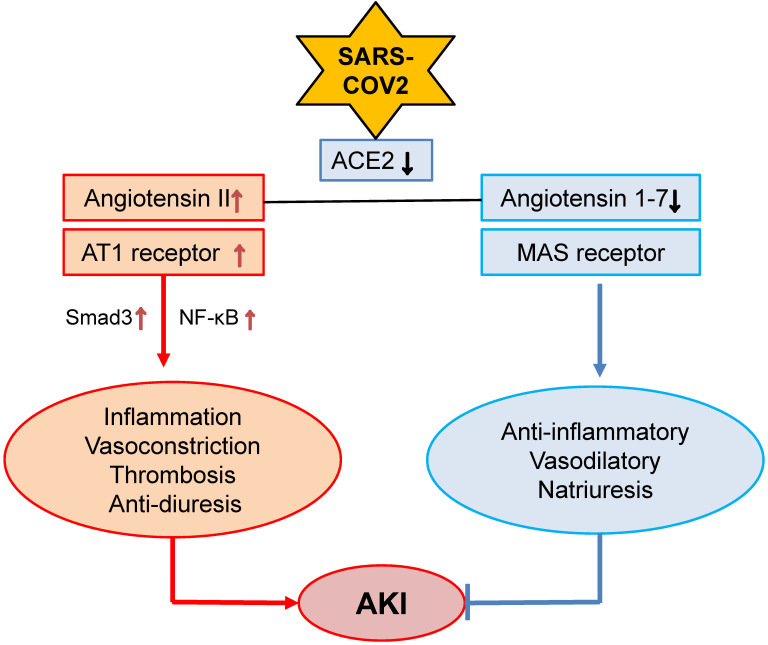
** Alterations of Ang II and Ang 1-7 signaling in COVID-19 associated AKI.** SARS-COV-2 binds and downregulates ACE2, which may result in downregulation of Ang 1-7 while upregulating Ang II-AT signaling to promote AKI.

Kidney is a target organ of SARS-COV-2 virus infection due to the high expression levels of angiotensin-converting enzyme 2 (ACE2), a receptor for SARS-COV-2 virus 24, in the kidney tissues, particularly in renal tubular epithelial cells (TECs) 25-27. Thus, SARS-COV-2 may be able to directly bind to ACE2 and infect kidney cells, which is supported by high levels of SARS-COV-2 spike (S) and nucleoprotein (N protein) in COVID-19 patients with AKI 9, 10, 28. In the kidney, renin-angiotensin-aldosterone system (RAAS) maintains renal hemodynamic and regulates renal sodium transport in both normal physiological states and pathological conditions. Ang II and Ang 1-7 are the two major effectors of RAAS and are tightly controlled by two major enzymes of ACE and ACE2 29. Ang II acts via its receptor-1 (AT1) to mediate renal inflammation and fibrosis by activating NF-kB and Smad signaling crosstalk pathways, whereas Ang 1-7 binds receptor Mas to counter-regulate these pathological effects of Ang II 29. The primary function of ACE2 is to covert Ang II to Ang 1-7 to exert its anti-inflammatory, vasodilatory and natriuretic properties 30 (**Figure 1**). After binding to ACE2, SARS-COV-2 significantly downregulates ACE2 expression 31, 32, resulting in a inhibition or loss of Ang 1-7 while enhancing Ang II-AT1-dependent renal inflammation, vasoconstriction, thrombosis and anti-diuresis effects 33 (**Figure 1**). It has been well documented that Ang II is a key mediator of AKI 34-36, whereas, the ACE2-Ang-1-7-Mas axis is renoprotective 37. Thus, SARS-COV-2 viral infection to the kidney may downregulate ACE2-Ang1-7-Mas signaling while promoting the Ang II-AT1 signaling to mediate renal inflammation and AKI. A similar mechanism is also found in patients with ARDS 38. Thus, the interaction between SARS-COV-2 virus and ACE2 may eventually impair the ACE2-Ang 1-7 while enhancing Ang II signaling, resulting in hypertension and inflammatory stress both systemically and locally in the kidney. This may well explain that hypertension is an independent risk factor in COVID-19 patients 39. However, the role of Ang II signaling in COVID-19 patients with progressive renal injury remains yet to be determined.

### Diabetes and metabolic stress

Diabetes is also a risk factor for AKI 40. Patients with diabetes are associated with the severity and death in pandemic influenza (H1N1) 41, SARS-COV 42 and MERS-COV 43. Recent studies also reported that COVID-19 patients with diabetes have higher AKI and mortality rate than those with non-diabetes 14, 44. This is also confirmed in a recent meta-analysis in 5497 COVID-19 patients 45.

It is now well accepted that metabolic stress including hyperglycemia, obesity, insulin resistance and high levels of glycosylation end products (AGEs) in patients with diabetes can trigger the production of pro-inflammatory cytokines and promotes the oxidative stress 46. Hyperglycemia is a risk factor for AKI in patients with diabetes 21. The IκB kinase-β (IKKβ)/NF-κB axis is a key inflammatory pathway in diabetes in response to hyperglycemia and insulin resistance 47. AGEs can also induce activation of NF-κB, resulting in production of pro-inflammatory cytokines 48. By comparing with non-diabetic COVID-19 patients, COVID-19 patients with diabetes have significantly higher levels of IL-6 and CRP 49. In addition, patients with diabetes also develop hypertension, which is associated with activation of Ang II-AT1 while inhibiting ACE2-Ang 1-7 signaling 50. In addition, since ACE2 is also expressed in pancreas, infection of SARS-COV-2 may also damage pancreatic islet β-cells and aggravate hyperglycemia 51. Thus, enhanced metabolic inflammation in the diabetic kidney may be another mechanism contributing to the development of AKI in COVID-19 patients. However, mechanisms responsible for the metabolic stress in AKI after COVID-19 infection remain yet to be explored.

### Cytokine storm

Inflammatory cytokines has been recognized as a critical factor in the progression of COVID-19 52, 53. The inflammatory response triggered by SARS-COV, MERS-COV or SARS-COV-2 can recruit and activate monocytes, macrophage and dendritic cells to produce inflammatory cytokines 54, 55, which may be essential in controlling the viral replication and cleaning the infected cells 56. However, overactive immune responses may cause excessive and persistent cytokine production that leads to cytokine storm and results in multiple organ dysfunction as seen in patients with severe SARS 57, MERS 58 and COVID-19 infection 59. In these patients, a number of proinflammatory cytokines such as interleukin (IL)-1β, IL-6, IL-12, interferon γ (IFN-γ) and monocyte chemoattractant peptide (MCP-1) are associated with extensive lung damage in SARS patients 60. The blood levels of IL-10, IL-15 and TGF-β1 are also positively correlated with the disease severity in patients with MERS 61. In COVID-19 patients, levels of IL-1β, IL-1RA, IL-7, IL-8, IL-9, IL-10, granulocyte colony stimulating factor (G-CSF), IFN-γ, interferon γ inducible protein (IP)-10, tumor necrosis factor-alpha (TNF-α) and MCP-1 are also increased over the healthy controls and become worsen in those admitted to ICU with severe acute lung injury 62, 63.

Cytokine storm can also trigger AKI under various clinical conditions including secondary haemophagocytic lymphohistiocytosis (sHLH) 64-68. sHLH is also found in patients with SARS and COVID-19 69, 70. In addition, cytokine storm may also result in the development of antiphospholipid syndrome in AKI patients with COVID-19 71, 72. Of these inflammatory cytokines, IL-6 has been recognized as a key mediator in COVID-19 patients, which is further described below.

#### IL-6

Many studies have demonstrated that among the inflammatory cytokines, IL-6 is a most strong and important mediator in COVID-19 patients 73, 74. Meta-analysis involving 12681 COVID-19 patients confirms that IL-6 is significantly higher in those with severe disease conditions 75, 76. Indeed, serum levels of IL-6 positively correlate with the severity of COVID-19 75, 77, 78 and also predict the mechanical ventilation need for COVID-19 patients 79. In COVID-19 patients with older age, IL-6 is an independent risk factor for in-hospital mortality 80.

**Figure 2:**
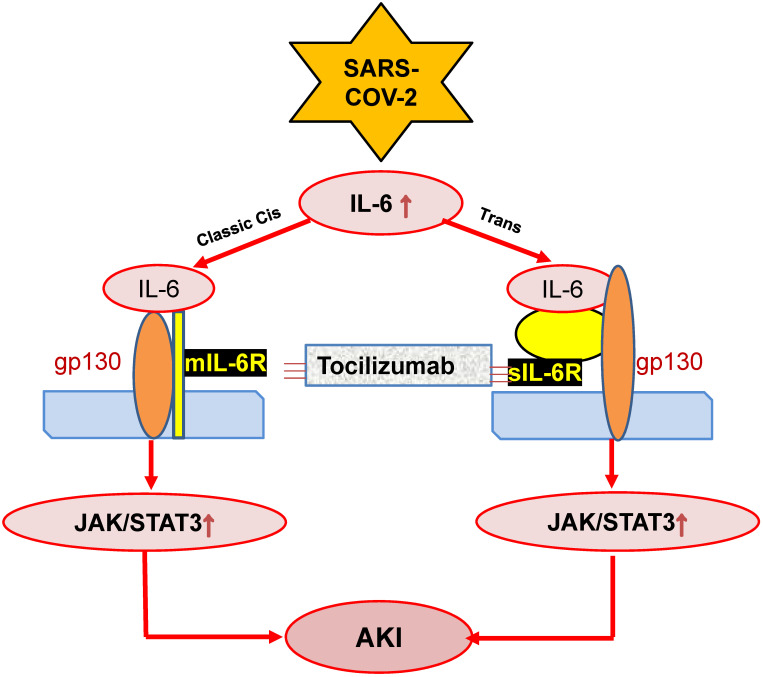
** Possible mechanisms through which SARS-COV-2 may induce AKI by stimulating IL-6 signaling.** SARS-COV-2 may activate IL-6-mIL-6/sIL6-JAK-STAT3 signaling, resulting in AKI, which can be blocked by Tocilizumab antibodies.

IL-6 is also a predictor for AKI in patients under various clinical conditions including cardiovascular disease, kidney diseases and liver transplantation 81-83. This is also found in ischemic AKI animal model 84. In response to injury, IL-6 is upregulated and released from renal TECs and plays an important role in the pathophysiology of AKI 85. Increasing evidence shows that IL-6 is not only a biomarker but also a mediator for AKI as mice lacking IL-6 are resistant to HgCl_2_-induced AKI 85-88. In patients with COVID-19, serum levels of IL-6 are elevated in those with AKI 89, and become further increased in those with critically ill 71, 90. In addition, serum levels of IL-6 can also predict the clinical outcomes of AKI as it is significantly reduced in those when AKI is recovered after effective treatment 91. Mechanistically, JAK-STAT3 is a downstream signal transduction of IL-6-membrane-bound-IL-6 receptor (mIL-6R)/soluble-bound-IL-6 receptor (sIL-6R). The IL-6-mIL-6R/sIL-6R-JAK-STAT3 signing pathways are activated during cytokine storm in severe COVID-19 patients 54, which is outlined in **Figure 2**. However, the functional role and molecular mechanisms of IL-6 in the pathogenesis of COVID-19 associated AKI remain largely unclear.

#### C-reactive protein (CRP)

**Figure 3:**
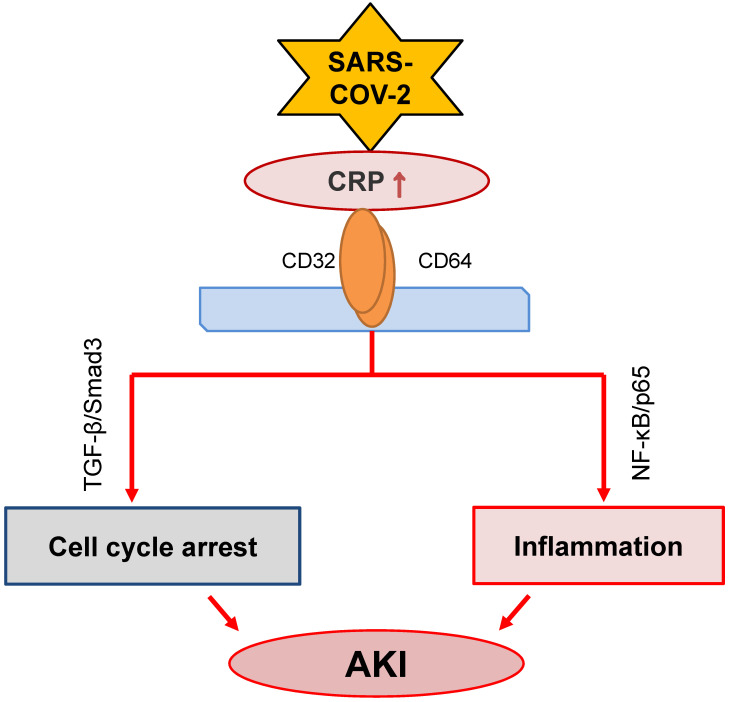
** Possible role of CRP signaling in COVID-19-associated AKI.** SARS-COV-2 infection may activate CRP signaling to cause AKI via TGF-β/Smad3-mediated G1 cell cycle arrest and NF-κB-dependent renal inflammation.

CRP, produced by liver and many inflammatory cells, is an acute phase protein. It has been widely used in clinical settings as an acute inflammation biomarker. CRP is proved as a predictor of postoperative AKI in patients undergoing Coronary Artery Bypass Graft (CABG) 92. High sensitive CRP is associated with AKI in patients with acute myocardial infarction 93, 94. Meanwhile, it is an independent predictor for AKI among ST elevation myocardial infarction patients undergoing primary percutaneous intervention 95. Increasing evidence has suggested that CRP is also a pathogenic factor contributing to the development of inflammatory diseases including atherosclerosis 96 and AKI 97-101. The mechanisms of CRP in the progression of AKI include stimulating macrophage activation 97, inducing cell death by causing G1 cell cycle arrest and autophagy 99, and promoting inflammation 101 (**Figure 3**). The activation of NF-κB/p65 and TGF-β/Smad3 signaling pathways are the major mechanisms through which CRP mediates AKI 98, 100, 101.

COVID-19 patients with AKI show higher levels of serum CRP over those without AKI 102. Serum levels of CRP are also a risk factor of AKI in COVID-19 patients 103. Tan et al. reported that serum levels of CRP are significantly elevated after SARS-CoV-2 infection, which becomes further increased when the disease is progressive but declines dramatically when COVID-19 is recovered 104. Thus, levels of serum CRP may be a predictor for the clinical outcomes of COVID-19 patients. Meta-analysis confirms this notion that in contrast to mild and survival subgroup of COVID-19 patients, high levels of CRP are associated with severe and death subgroup of COVID-19 patients 75, 76, 105. In addition, CRP is also an indicator for renal replacement therapy and the need for mechanical ventilation in COVID-19 patients 106. Thus, elevated CRP is independently associated with poor clinical outcomes in COVID-19 patients 107, 108. However, the pathogenic role and mechanisms of CRP in COVID-19-associated AKI remain largely unknown.

#### TGF-β

TGF-β is a pleiotropic cytokine and signals through its downstream canonical and non-canonical pathways to diversely regulate renal inflammation and fibrosis 109, 110. It has been reported that SARS-COV nucleocapsid protein can interact with Smad3 to activate the canonical pathway 111. Whereas, the non-canonical TGF-β signaling pathway is also activated by the papain-like protease of SARS-COV by inducing expression of TGF-β1 112. In COVID-19 patients, plasma levels of TGF-β are significantly elevated and associated with the disease severity and poor clinical outcomes 113, 114. Elevated TGF-β2 mRNA is also found in the bronchoalveolar lavage (BAL) fluid of COVID-19 patients 115, which may contribute to lung inflammation and fibrosis because TGF-β1 is also a growth factor associated with fibrosis [109.110]. It is reported that SARS-COV-2 encoded microRNAs are able to target TGF-β signaling pathway to induce TGF-β-dominated adaptive immune response 116. Upregulation of TGF-β1 in COVID-19 patients is responsible for the recruitment of neutrophils into the site of inflammation 117. TGF-β can also induce MCP-1 to activate macrophage-dependent inflammation in the diabetic kidney via a Smad3-dependent LRNA9884 118. Induction of IL-6 production by TGF-β1 also leads to systemic inflammation and “cytokine storm” 119. Furthermore, TGF-β can induce IgA class switching, which may contribute to vasculitis in critically ill patients with COVID-19 120. Thus, TGF-β may significantly contribute to the immediate and long-term effects of COVID-19.

**Figure 4:**
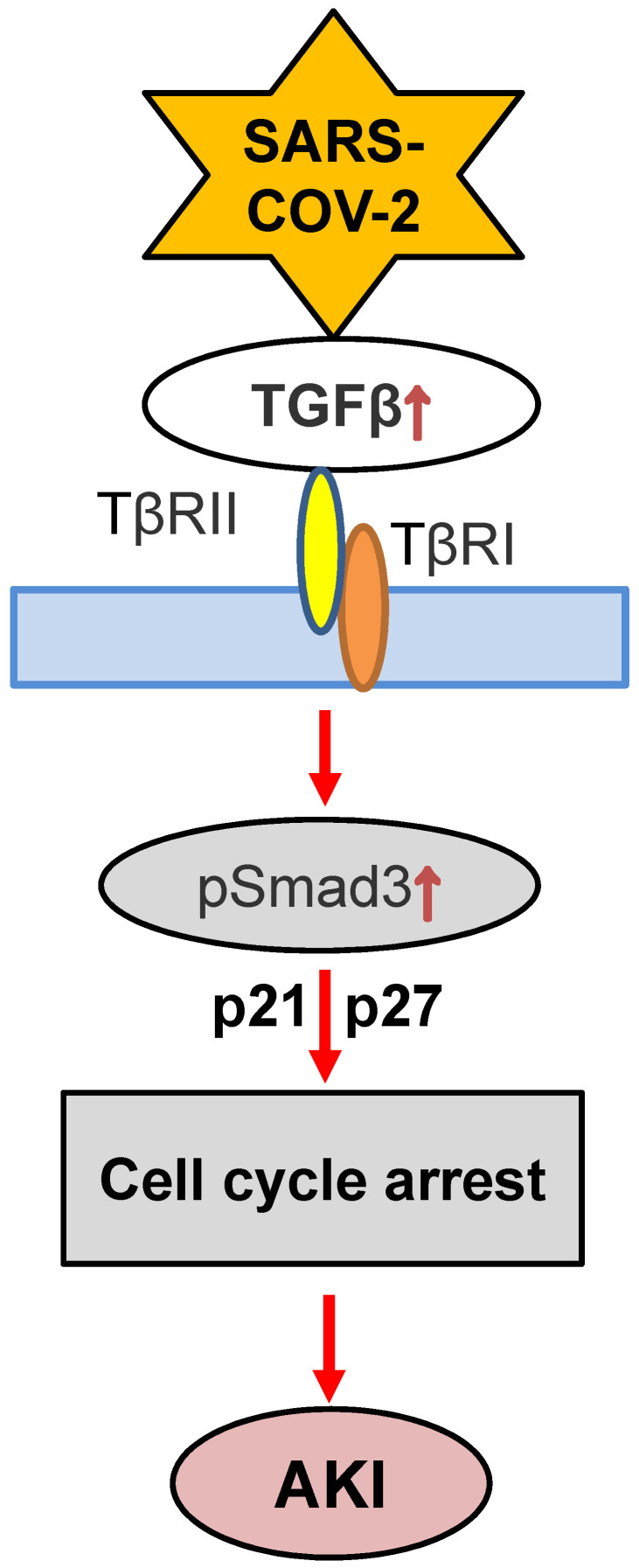
** Proposed TGF-β signaling in COVID-19-associated AKI.** SARS-COV-2 may induce G1 cell cycle arrest and cell death via TGF-β/Smad3-p21/p27 mechanism.

Although the role of TGF-β in COVID-19 associated AKI is still unclear, recent findings that conditional deletion of TGF-β receptor II from renal proximal tubules protects against mercuric chloride and cisplatin-induced AKI reveal a critical role of TGF-β signaling in AKI 121, 122. Furthermore, mice specifically lacking bronchial epithelial TGF-β1 (epTGFβKO) are protected against influenza-induced weight loss, airway inflammation, and viral replication 123, suggesting the impact of TGF-β in viral infection. Mechanistically, TGF-β1 may act via Smad3 to cause AKI as genetic deletion or pharmacological inhibition of Smad3 can block AKI in ischemic mice with or without high human CRP conditions 124. Indeed, Smad3 can be activated by both TGF-β-dependent and independent mechanisms including Ang II, advanced end products (AGE), and CRP under various disease conditions such as hypertension and diabetes 125-127. Thus, activation of Smad3 signaling may account for the clinical notion that patients with diabetes and hypertension are high risk for COVID-19 7. Mechanistically, Smad3 promotes AKI by directly binding to p21/p27 to suppress CDKs/cyclin E to cause the G1 cell cycle arrest 128, 129 (**Figure 4**). Thus, it is highly possible that TGF-β/Smad3 signaling may contribute to COVID-19-associated AKI, which is warranted for further investigation.

### Complement activation

The complement system is the first response of host immune system that recognizes and eliminates virus, such as SARS-COV or SARS-COV-2 7, 130. There are several pathways involving in systemic complement activation including the lectin pathway, classical pathway, and alternative pathway 130, 131. The lectin pathway is triggered by the binding of mannose-binding lectin (MBL) with SARS-COV spike (S) protein 132, which leads to the activation of mannan-binding lectin-associated serine protease 2 (MASP-2). The N protein of coronavirus is also associated with the severity of lung injury according to MASP-2-mediated complement overactivation. Thus, alteration of MASP-2-binding motif or blocking the MASP-2-N protein interaction attenuates lung injury 133. The classical pathway is activated by the binding of antibodies, which forms the immune complexes with viral antigens to complement C1 complex 130, 131. The classical, lectin and alternative pathways resulting in the formation of C3 convertase to activate the complement system, which is observed in the lung after SARS-COV infection 130, 131, 134. The role of C3 in SARS-COV-induced lung injury is confirmed in C3 deficient mice in which deletion of C3 protects against ARDS with lower levels of cytokine and inflammatory monocytes infiltration 135.

The complement system is activated during the progression of COVID-19 136. The activation of complement system is related to the disease severity and the respiratory failure in COVID-19 patients 134, 136, 137. It has been shown that the C5a-C5aR1 axis plays an important role in the development of ARDS in COVID-19 138, whereas, C4d and C5-9 are colocalized with the SARS-COV-2 S protein in the lung and skin vasculature 139.

The activation of alternative pathway of complement also participates in the pathogenesis of AKI 140, 141. It has been reported that local synthesis or deposition and activation of complement by renal epithelium is an important cause of AKI 142, 143. In addition, strong C5b-9 staining has been demonstrated on the apical brush border of TECs of the kidney with SARS-COV-2 infection 10. Activation of the classical complement pathway is also observed in the AKI kidney in critically ill children with COVID-19 90. However, the functional role and mechanisms of complement activation in COVID-19-assicated AKI remain largely unclear.

### The lung-kidney crosstalk pathway

The crosstalk between the lung and kidney has been observed in patients with AKI and ARDS 144. Clinically, acute lung injury and AKI are complications often encountered in patients with critical illness 145. Mechanical ventilation can improve lung function but is a risk factor for AKI in critically ill patients 146, 147. It has been reported that positive pressure of mechanical ventilation can increase the risk of AKI by almost 8-fold 106, which is associated with systemic hemodynamic and neurohormonal changes and biotrauma 65, 148, 149. ARDS can trigger AKI via mechanisms associated with systemic hypoxia, hypercapnia, systemic inflammatory response syndrome (SIRS), and mechanical ventilation. Severe hypoxemia in ICU patients is associated with AKI required for renal replacement therapy 145. Kidney is susceptible to hypoxic injury due to the high rate of oxygen consumption 65, 150. Thus, hypoxia can induce AKI and tubular necrosis or apoptosis 149. Hypercapnia in COVID-19 patients can also affect the renal blood flow by stimulating renal vasoconstriction 151. More importantly, the lung-kidney cross-talk is also associated with the cytokine storm 152. The inflammatory reaction caused by the lung injury can damage the kidney to release abundant inflammatory cytokines, which, in turn, promotes the damage in the lung 8. Thus, the approaches to limit ventilator-induced lung injury and decrease the duration of mechanical ventilatory support to protect against AKI in critically ill patients are proposed for treatment of critically ill COVID-19 with acute lung and kidney injury.

## Therapeutic potential for COVID-19-associated AKI

### Continuous renal replacement therapy (CRRT)

CRRT is an advanced approach to treat patients with AKI by improving overload water status and removing inflammatory factors 153, particularly in those with septic AKI 154. CRRT has been utilized in AKI patients with severe MERS and in critically ill COVID-19 155. However, whether the early initiation or high intensity of CRRT can improve the progression of AKI COVID-19 patients remains to be determined.

### Tocilizumab (TCZ)

TCZ, a recombinant humanized monoclonal antibody against the human membrane and soluble IL-6 receptors, is widely used for treatment of immunoinflammatory rheumatic diseases 156, 157. TCZ has been shown to block the IL-6/NF-κB/JNK pathway to have a protective effect against sepsis-induced acute lung injury and AKI 158. As IL-6 is important in COVID-19, TCZ has been used to treat COVID-19 patients clinically (**Figure 2**). The early treatment with TCZ has been shown to effectively improve the oxygen status in COVID-19 patients 156. The meta-analysis of TCZ studies in a total of 1675 and 6279 COVID-19 patients with critically ill shows that TCZ treatment can significantly reduce the in-hospital mortality rate, although patients remain the need for hemodialysis and ventilation 159. Thus, the therapeutic efficacy of TCZ on COVID-19-associated AKI needs to be further studied.

### Complement inhibitor

#### A C3 inhibitor AMY-101

AMY-101 is a highly selective and potent C3 inhibitor and is currently tested in Phase II clinical trials in patients with sepsis, hemodialysis-induced inflammation or malarial anemia 160-163. It is reported that treatment with AMY-101 is safe and can significantly improve the clinical manifestations in severe COVID-19 patients 164. Further Phase II and III clinical trials are still going.

#### An anti-C5 antibody

An anti-C5 antibody has been clinically used in patients with C3 glomerulopathy and several types of AKI including atypical hemolytic uremic syndrome and paroxysmal nocturnal hemoglobinuria 165. Treatment with the anti-C5 antibody has shown to improve the kidney function and ameliorate the intra-renal complement activation and systemic inflammation in ischemia reperfusion-induced AKI mouse model 165. The first result of anti-C5 treatment has also revealed a rapid and promising effect on COVID-19 patients 166. However, more clinical trials are needed for the conclusive results of the anti-C5 antibody treatment on COVID-19 patients with AKI.

#### Anti-TGF-β treatment

As SARS coronavirus can upregulate TGF-β and patients with coronavirus infection have elevated levels of TGF-β 113, 114, it has been proposed that TGF-β could be a valid target for the treatment of COVID-19 167, 168. In a recent Phase II clinical trial, inhibition of TGF-β expression by OT-101, an anti-sense to TGF-β1, has been shown to suppress SARS-COV and SARS-COV-2 replication and allow patients to recover without going into respiratory crisis 169, suggesting that COVID-19 can be treated with TGF-β inhibition. However, it should be noted that TGF-β has diverse roles in renal inflammation and fibrosis 109, 110 and targeting the upstream of TGF-β may also cause adverse effects. Our recent studies showed that TGF-β can trigger AKI via the Smad3-dependent mechanism and treatment with Smad3 inhibitors such as SIS3 or a natural product of Traditional Chinese Medicine Quercetin can effectively suppress AKI even under high human CRP conditions 100, 170. These findings suggest that targeting Smad3 specifically, rather than the entire TGF-β signaling, may represent as a novel and effective therapy for AKI in COVID-19 patients clinically.

## Conclusion

AKI is a common complication in critically ill COVID-19 patients. Inflammation may be a key mechanism triggering this process. Many inflammatory stress molecules and pathways including Ang II-associated hypertensive stress, diabetes-related metabolic stress, cytokine storm, high CRP, overreactive TGF-β signaling, complement activation, and lung-kidney crosstalk may promote AKI in COVID-19 patients. Thus, treatments by targeting these molecules and pathways may represent as a novel and specific therapy for AKI in COVID-19 patients.

## Abbreviations

ACE2: angiotensin-converting enzyme 2
Ang I: Angiotensin II
AKI: Acute kidney injury
ARDS: acute respiratory distress syndrome
BUN: Blood urea nitrogen
CRP: C-reactive protein
CABG: Coronary Artery Bypass Graft
CRRT: Continuous renal replacement therapy
G-CSF: granulocyte colony stimulating factor
IFN-γ: interferon γ
IKKβ: IκB kinase-β
IL-6: Interleukin-6
IP: inducible protein
MASP-2: mannan-binding lectin-associated serine protease 2
MCP-1: monocyte chemoattractant peptide
mIL-6R: membrane-bound-IL-6 receptor
RAAS: renin-angiotensin-aldosterone system
Scr: serum creatinine
sIL-6R: soluble-bound-IL-6 receptor
TCZ: Tocilizumab
TNF-α: tumor necrosis factor-alpha
